# Physicochemical Quality and Chemical Safety of Commercial Wet Cat Food: Composition, Oxidation, Biogenic Amines, and Trace Elements

**DOI:** 10.1111/jpn.70080

**Published:** 2026-05-27

**Authors:** Marina Teixeira de Vries Mársico, Eliane Teixeira Mársico, Celso Fasura Balthazar, João Paulo de Sá Felizardo, Roberto Meigikos dos Anjos, Erick Almeida Esmerino, Sérgio Borges Mano

**Affiliations:** ^1^ Department of Food Technology (MTA), Veterinary School Federal Fluminense University Niterói Rio de Janeiro Brazil; ^2^ Laboratory of Radioecology and Environmental Change (LARA), Institute of Physics Federal Fluminense University Niterói Rio de Janeiro Brazil

**Keywords:** feed safety, histamine, lipid oxidation, mercury, pet food, protein carbonylation

## Abstract

This study investigated the physicochemical characteristics and chemical safety of commercial wet cat foods marketed in Brazil. Twenty products labeled as beef, chicken and fish flavors were analyzed for proximate composition, pH, lipid and protein oxidation, biogenic amines, and essential and potentially toxic trace elements. Moisture ranged from 78.13% to 94.87%, and protein varied from 37.38% to 89.97% dry matter. Thiobarbituric acid reactive substances (TBARs) value reached 2.49 mg malondialdehyde (MDA)/kg dry matter, and carbonyl protein up to 0.81 nmoL/mg protein dry matter, indicating oxidative damage in part of the samples. Histamine, putrescine and cadaverine were detected on average levels of 7.5, 3.5 and 3.5 mg/kg wet matter, respectively, indicative for microbial activity and/or the use of raw materials of suboptimal quality. Trace element analysis revealed mercury concentration between 0.00 and 1.34 mg/kg, Cu (0.01 mg/kg) and Zn (45−50 mg/kg) were found in some samples (dry matter). The evidence of oxidation, biogenic amine and toxic elements in wet pet food, even within available international reference values, is a warning for chronic consumption by cats, a species with limited hepatic detoxification capacity. Overall, the findings highlight the need for strengthened quality assurance schemes and systematic surveillance on wet pet foods to better safeguard feline health.

## 1 Introduction

The global pet food market has grown rapidly, driven by the humanization of companion animals and the demand for nutritionally optimized diets (Kumar et al. 2024). Cats represent a particularly demanding species due to their strict obligate carnivory, high and continuous protein requirements, distinctive amino acid and nitrogen metabolism, dependence on preformed nutrients (taurine, arachidonic acid, retinol, vitamin D), and limited hepatic detoxification capacity (Verbrugghe and Hesta 2017). Because their liver possesses a reduced ability to perform glucuronide conjugation, a vital Phase II detoxification process that converts toxins into water‐soluble substances for excretion in urine or bile (Otte et al. 2017). These physiological traits make cats highly susceptible to nutritional imbalances, oxidative degradation products, and chemical contaminants in commercial diets (Che et al. 2021).

Wet cat food is increasingly popular because of its high palatability and moisture content (Le Guillas et al. 2024). Unlike dry kibble, which typically contains less than 10% moisture, wet diet high moisture formulations provide a significant proportion of a cat's daily water requirements directly through food consumption (Da Silva et al. 2022). This is particularly relevant for felines, as they possess a naturally low thirst drive and do not adequately compensate for dry diet low moisture by increasing voluntary water intake (Buckley et al. 2011). Consequently, the consumption of wet diets promotes higher hydration status and is associated with a reduced risk of lower urinary tract diseases, including urolithiasis and feline idiopathic cystitis (Groves 2021; Zanghi et al. 2018).

However, the quality and safety of wet cat food depend on both industrial processing and post‐opening handling. When properly manufactured, retort sterilization and hermetic packaging ensure stability throughout the unopened shelf life (Raits et al. 2021). Conversely, suboptimal formulation, processing, or packaging integrity may lead to degradation even before opening. Once opened, the product becomes vulnerable to the following main categories of degradation. Biotic degradation is caused by microbial growth, including bacteria, molds, and yeasts, which thrive due to the high‐water activity (> 0.85) and nutrient‐rich matrix of wet food. This microbial activity leads to the accumulation of biogenic amines such as histamine, putrescine, and cadaverine, which serve as indicators of spoilage and may pose health risks to cats, including allergic reactions and interference with metabolic functions (Altafini et al. 2022; Mafe et al. 2024). Abiotic degradation results from non‐biological chemical reactions, primarily lipid and protein oxidation. Exposure to residual oxygen, light, or temperature fluctuations triggers the formation of malondialdehyde (MDA) and protein carbonyls, which reduce nutritional value, generate off‐flavors, and may contribute to chronic inflammatory conditions in felines (Geng et al. 2023). If not properly stored under refrigeration and consumed within the recommended timeframe, these biotic and abiotic processes accelerate rapidly. Consequently, oxidation products, biogenic amines, and trace element contaminants may act as subtle but cumulative risk factors for feline health—risks that are frequently underestimated in veterinary practice and regulatory frameworks (Varrà et al. 2025).

Recent literature emphasizes the importance of evaluating pet foods beyond proximate composition by including markers of oxidative stability, biogenic amine profiles (indicators of raw material freshness, hygiene, and proteolysis), and concentrations of essential and potentially toxic trace elements (notably Hg, As and Pb) (Haydous et al. 2025; Varrà et al. 2025). These contaminants often originate from fish, fish by‐products, and animal viscera (liver and kidneys) due to environmental bioaccumulation or deficiencies in sourcing and processing (Özden et al. 2024; Wei et al. 2025).

In Brazil and broader South America, peer‐reviewed data on the physicochemical quality, oxidative status, biogenic amines, and trace element contamination of commercial wet cat foods remain scarce, and specific regulatory limits for many chemical contaminants in pet foods are lacking (Zafalon et al. 2021). Despite updates from FDA enforcing regulatory limits for chemical contaminants in pet foods, such as micotoxins, pesticides and others (US Food and Drug Administration FDA 2025), still remain knowledge and regulatory gaps, which are concerning for several reasons. First, cats are obligate carnivores with a limited capacity for hepatic detoxification, making them uniquely vulnerable to chronic, low‐level exposure to contaminants such as mercury, arsenic, and biogenic amines, which pose a risk with repeated exposure (Du et al. 2025). Second, the high moisture content and complex protein‐lipid matrix of wet foods create an environment conducive to oxidative degradation and biogenic amine formation when processing or storage conditions are suboptimal (Geng et al. 2023). Third, without established regulatory limits or systematic surveillance programs, manufacturers lack clear benchmarks for quality control, veterinarians cannot adequately counsel clients on product selection, and consumers remain unaware of potential risks associated with long‐term feeding of suboptimal products (Khuzina et al. 2025). Consequently, the absence of comprehensive quality data impedes the development of evidence‐based nutritional recommendations and undermines efforts to ensure the safety and nutritional adequacy of commercial wet cat foods in the Brazilian market.

Therefore, the present study aimed to comprehensively assess the nutritional and chemical quality of commercial wet cat foods available on the Brazilian market. Proximate composition, pH, lipid and protein oxidation markers, biogenic amine profiles, and concentrations of essential and potentially toxic trace elements were determined across different product categories and flavor types. By integrating these compositional, oxidative (lipids oxidation: thiobarbituric acid reactive substances [TBARs]; and carbonyl protein), and contaminant indicators, this work seeks to provide scientific evidence to support regulatory improvement, strengthen industry quality control, and inform evidence‐based nutritional recommendations for feline health.

## 2 Materials and Methods

### 2.1 Sample Selection and Classification

A total of 20 commercially available wet cat food pouches (85 g) from different manufacturers were selected from major retail outlets in Brazil between 2023 and 2024. All samples shared the same shelf‐life period (produced in the same month) and were acquired a month of fabrication. The samples were categorized as labeled (beef, chicken, or fish flavor, Table 1). All products were intended for adult cats and acquired in their original, sealed packaging within the labeled shelf life. These pouches were hermetically sealed, allowing for long‐term shelf stability at room temperature without the need for preservatives (Raits et al. 2021).

**Table 1:** Declared ingredients of commercial wet cat food samples according to their quantity.

Sample	Declared Ingredients
B1	Meat and meat by‐products (beef, poultry, liver), water, gelling agents, minerals, vitamins, taurine
B2	Poultry by‐products, beef, water, liver, gelling agents, minerals, vitamins, taurine, coloring agents
B3	Beef, poultry by‐products, water, liver, gelling agents, minerals, vitamins, taurine
B4	Poultry by‐products, beef, water, liver, gelling agents, minerals, vitamins, taurine
B5	Beef, poultry by‐products, water, liver, minerals, vitamins, taurine, gelling agents
B6	Poultry by‐products, beef, water, liver, gelling agents, minerals, vitamins, taurine. No artificial colors, flavors, or synthetic preservatives.
B7	Poultry by‐products, beef, water, liver, gelling agents, minerals, vitamins, taurine. No artificial colors, flavors, or synthetic preservatives.
C1	Poultry by‐products, chicken, water, liver, gelling agents, minerals, vitamins, taurine, coloring agents
C2	Chicken, poultry by‐products, water, liver, gelling agents, minerals, vitamins, taurine
C3	Poultry by‐products, chicken, water, liver, gelling agents, minerals, vitamins, taurine
C4	Chicken, poultry by‐products, water, liver, gelling agents, minerals, vitamins, taurine, coloring agents
C5	Poultry by‐products, chicken, water, liver, whole wheat flour, gelling agents, minerals, vitamins, taurine
C6	Chicken, chicken liver, water, whole wheat flour, gelling agents, minerals, vitamins, taurine. No artificial colors, flavors, or synthetic preservatives.
C7	Chicken, poultry by‐products, water, liver, gelling agents, minerals, vitamins, taurine. No artificial colors, flavors, or synthetic preservatives.
F1	Fish (tuna), poultry by‐products, water, gelling agents, minerals, vitamins, taurine, coloring agents
F2	Poultry by‐products, fish, water, liver, gelling agents, minerals, vitamins, taurine
F3	Fish, poultry by‐products, water, liver, gelling agents, minerals, vitamins, taurine
F4	Poultry by‐products, fish, water, liver, gelling agents, minerals, vitamins, taurine
F5	Tuna, fish broth, poultry by‐products, water, gelling agents, minerals, vitamins, taurine
F6	Fish, poultry by‐products, water, liver, gelling agents, minerals, vitamins, taurine. No artificial colors, flavors, or synthetic preservatives.

*Note:* *Pouches flavored as beef (B), chicken (C) or fish (F).

Pouch material of all samples was made from multi‐layer laminates, commonly combining an outer PET layer for printing, a middle aluminum foil layer for oxygen/light barrier protection, and an inner, heat‐resistant food‐grade polypropylene layer. As well, all packages were intact, free from swelling or leakage. Based on batch code information, all samples were stored under refrigeration (4 ± 1°C) and analyzed after 48 h acquisition. Each pouch unit was homogenized using a sterile stainless‐steel blender to obtain a representative composite for analyses.

### 2.2 Proximate Composition

Moisture, protein, lipid, and ash contents were determined according to the AOAC International (2019) official methods. A gravimetric method (AOAC 925.10) for moisture was performed by drying at 105°C until constant weight. Protein was carried out by Kjeldahl method (AOAC 984.13) using a nitrogen to‐protein conversion factor of 6.25. Lipids were acquired by Soxhlet extraction (AOAC 991.36) with petroleum ether. Ash was measured after incineration at 550°C (AOAC 942.05). As well, total carbohydrates were estimated by difference with other components. Moisture was expressed on a wet matter basis (%). However, nutrient content was expressed on dry matter basis (DMB) as: Nutrient_DMB_ = 100 × (nutrient_wet basis_/100–moisture).

### 2.3 Lipid and Protein Oxidation

Lipid peroxidation was evaluated using the TBARs method, based on Tarladgis et al. (1960) with modifications. Approximately 10 g of each sample was homogenized with trichloroacetic acid (TCA, 7.5%) and distilled. The distillate was reacted with 0.02 M TBA and heated at 100°C for 35 min. Absorbance was measured at 532 nm using a UV‐Vis spectrophotometer (Shimadzu, Japan). Results were expressed as mg MDA per kg of sample on a wet basis, using a standard curve of 1,1,3,3‐tetraethoxypropane. To enable comparison across products with variable moisture content, TBARS values were also calculated on a DMB using the formula: TBARS_DMB_ = TBARS_wet_ × (100/(100−moisture)).

Protein oxidation was assessed by quantifying carbonyl groups through reaction with 2,4‐dinitrophenylhydrazine (DNPH), following Requena et al. (2003). Proteins were extracted in phosphate buffer (20 mM, pH 6.5) and reacted with 10 mM DNPH in 2 M HCl for 1 h at room temperature in the dark. After precipitation with 20% TCA and washing with ethanol/ethyl acetate (1:1), the pellets were solubilized in 6 M guanidine hydrochloride and absorbance was measured at 370 nm using a UV‐Vis spectrophotometer. Carbonyl content was calculated using the molar extinction coefficient ε = 22,000 M⁻¹ cm⁻¹ and expressed as nmol carbonyl/mg protein. As protein carbonyls are already normalized to protein content, no further moisture‐based normalization was required.

### 2.4 pH

The pH of each sample was determined using a calibrated digital pH meter (Hanna HI2211) with a penetration electrode, inserted directly into the homogenized sample at room temperature.

### 2.5 Biogenic Amines

The presence of the biogenic amines histamine, putrescine, and cadaverine was assessed using thin‐layer chromatography (TLC), following the method of Schutz et al. (1976) with in‐house adaptations and validation. Extracts from food samples and standard solutions were applied to silica gel 60 F₂₅₄ plates (Merck, Germany).

Migration was performed in a saturated chamber using the solvent system methanol: 25% ammonium hydroxide (4:1, v/v). Amines were visualized by spraying with 0.2% ninhydrin reagent in acetone, followed by heating at 110°C for 10 min.

Identification and concentration estimation were based on the direct comparison of the color intensity and retention factor (Rf) of sample spots with those of a calibration curve run on the same plate, using standard amine solutions at known concentrations (0, 1, 2, 5, 10, 20 mg kg⁻¹). To minimize subjectivity, reading was performed independently by two trained analysts, achieving an inter‐rater agreement of 92% (Cohen's Kappa = 0.85), considered excellent. The visual Limit of Detection (LOD) of the method was established at 1 mg kg⁻¹ for all amines.

Based on this semi‐quantitative comparison, each sample was classified into one of the following pre‐defined concentration ranges:

- Histamine: Low (< 5 mg kg⁻¹), Moderate (5 – < 10 mg kg⁻¹), High (≥ 10 mg kg⁻¹).
- Putrescine and Cadaverine: Moderate (2 – < 5 mg kg⁻¹), High (≥ 5 mg kg⁻¹). Concentrations below 2 mg kg⁻¹ were recorded as “< 2 mg kg⁻¹” and treated as censored data for statistical analyses.

To enable a robust statistical comparison between groups (flavors: beef, chicken and fish), the original categorical data were subjected to a numerical modeling process, whose limitations are acknowledged and explicitly stated. Each concentration range was converted into a single representative numerical value using the midpoint of the range rule. This rule was chosen as it is the least biased central estimate for an interval. The assigned values were:

- Histamine: Low = 2.5 mg kg⁻¹; Moderate = 7.5 mg kg⁻¹; High = 15.0 mg kg⁻¹ (considering 10 mg kg⁻¹ as the lower limit of a representative upper range).
- Putrescine/Cadaverine: Moderate = 3.5 mg kg⁻¹; High = 7.5 mg kg⁻¹. Samples below the LOD (< 2 mg kg⁻¹) were assigned a value of 1.0 mg kg⁻¹ for calculation purposes, but their results were interpreted with caution.

To assess the robustness of the conclusions regarding the assignment rule, a sensitivity analysis was conducted in parallel by repeating the statistical tests using the lower and upper limits of each range.

### 2.6 Trace Elements

The determination of total mercury (Hg) was performed by cold vapor atomic absorption spectrometry (CV‐AAS), based on the US EPA Method 7473. Samples (wet basis) were weighed directly into quartz boats and introduced into a direct mercury analyzer (DMA‐80, Milestone), which operates via thermal decomposition, catalytic conversion, amalgamation, and atomic absorption detection.

This method does not require acid digestion and is highly sensitive for mercury quantification in biological matrices. Calibration was conducted using certified mercury standards, and quality control was ensured through analysis of blanks and certified reference material (DORM‐4, fish protein). Results were expressed in mg Hg/kg of wet sample. The method detection limit (MDL) was 0.001 mg/kg. To enable comparison across products with variable moisture content, mercury concentrations were also calculated on DMB using the formula: Hg_DMB_ = Hg_wet_ × (100/(100−moisture)).

The determination of total concentrations of essential (Cu, Zn) and potentially toxic (As, Pb) trace elements was performed using wavelength‐dispersive X‐ray fluorescence spectrometry (WDXRF). Approximately 5 g of each sample (previously lyophilized and homogenized) was manually blended with 0.5 g of Spectra binder, then pressed into 25 mm pellets using a tungsten matrix mold and a manual X‐Press 3636 SpexSamplePrep press applying 20 tons for 3 min, following the preparation protocol described by Marguí et al. (2005). This procedure ensured the formation of compact, uniform discs suitable for X‐ray analysis.

**Figure 1:**
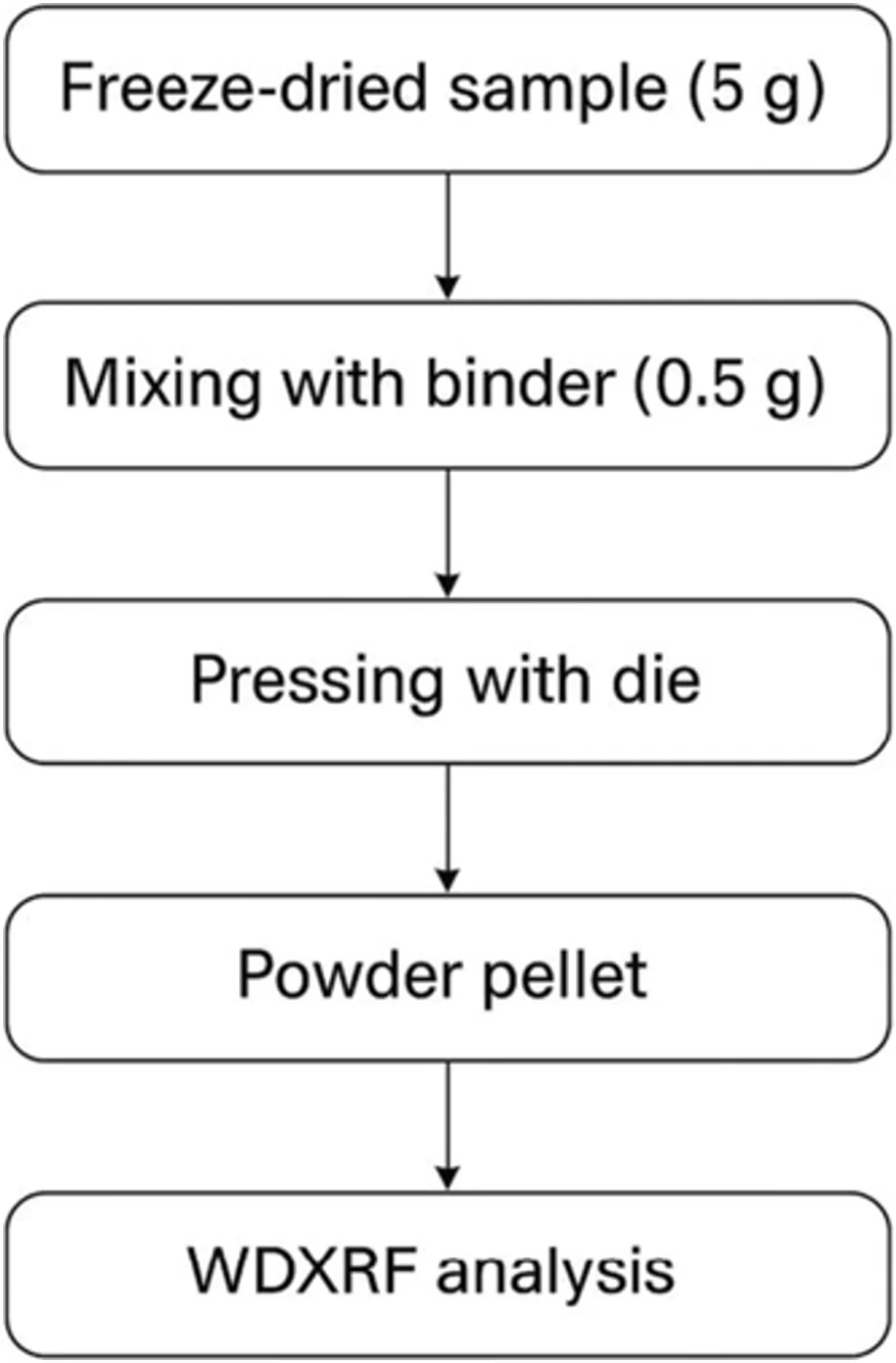
Wet cat food sample preparation for WDXRF.

The analyses were conducted using a Rigaku SUPERMINI 200 WDXRF spectrometer, equipped with a 200 W X‐ray tube and a multi‐crystal changer, enabling high sensitivity detection and precise wavelength dispersion. This non‐destructive technique allows for accurate quantification of trace elements in complex food matrices without the need for acid digestion or sample combustion, offering excellent repeatability and matrix compatibility (Figure 1).

The results were expressed in mg/kg (DMB). The method has been validated in the literature for food analysis (Frydrych and Jurowski 2023) and was calibrated using matrix‐matched pressed‐powder standards with certified concentrations.

### 2.7 Statistical Analysis

All analyses were performed in six replications of each sample for each wet cat food pouch sample (*n* = 20). Data were analyzed by one‐way ANOVA followed by Tukey's post hoc test, with the significance level set at *p* < 0.05. Differences among flavor groups (beef, chicken, and fish) were evaluated as independent factors. As each of the 20 samples originated from a different manufacturer, manufacturer was not included as a factor in the ANOVA.

The transformed TLC data were analyzed considering their ordinal and non‐parametric nature. Central tendency and dispersion were presented as median and interquartile range (IQR; Q1−Q3). For comparison among the three flavor groups (beef, chicken and fish), the Kruskal‐Wallis test was used, followed by Dunn's test with Bonferroni correction for post hoc multiple comparisons. The significance level was set at *p* < 0.05. All analyses were performed using R software (v.4.3.1).

## 3 Results and Discussion

### 3.1 Proximate Composition in Commercial Wet Cat Food

The proximate composition analysis of commercially available wet cat foods revealed significant quantitative differences in moisture, protein, lipid, ash, and carbohydrate contents among and within the beef, chicken, and fish‐flavored product categories (*p* < 0.05). As detailed in Table 2, moisture was the most variable component, ranging from 78.13% to 94.87% (*p* < 0.05). While the overall moisture mean was higher in the fish‐flavored group (80.18% to 88.05%). The wide variation in moisture for wet pet food is primarily driven by the high‐water content of raw ingredients like meat and offal that naturally contain between 57% and 75.25% moisture (Sires et al. 2019). Because moist pouch diets do not require the high starch levels (often > 30%) necessary for the expansion and texturization of dry kibble, manufacturers can incorporate significantly more liquid using gelling agents and thickeners such as sodium alginate or carrageenan (Sgorlon et al. 2022). While commercial wet foods typically declare a maximum moisture of around 78%, home‐prepared diets often exceed these levels because they utilize fresh ingredients without industrial dehydration (Sires et al. 2019).

**Table 2:** Commercial wet cat food composition: moisture (% original matter) and nutrient content (% dry matter basis).

Sample	Moisture (%)	Protein (%)	Fat (%)	Ash (%)	Carbs (%)
B1	82.32 ± 0.80^e,f,g^	51.15 ± 0.82^d,e,f^	17.94 ± 0.11^e,f^	17.21 ± 0.11^c^	13.70 ± 0.70^e^
B2	81.25 ± 1.06^f,g^	40.77 ± 0.82^h,i,j^	20.78 ± 0.51^d^	15.69 ± 0.21^d^	22.76 ± 1.32^c^
B3	84.25 ± 0.76^d,e^	49.30 ± 0.43^e,f,g^	18.94 ± 0.18^d,e^	9.12 ± 0.20^j,k^	22.64 ± 0.87^c^
B4	89.28 ± 0.44^b^	89.97 ± 0.97^a^	10.00 ± 0.19^h^	8.92 ± 0.12^j,k^	0.00 ± 0.00^i^
B5	84.05 ± 1.09^d,e^	50.13 ± 0.44^d,e,f,g^	25.45 ± 0.43^b^	25.30 ± 0.30^a^	2.78 ± 0.73^h,i^
B6	86.53 ± 0.61^b,c,d^	58.81 ± 0.67^b^	7.09 ± 0.08^i^	21.41 ± 0.22^b^	12.86 ± 0.59^e^
B7	87.80 ± 0.41^b,c^	57.56 ± 0.82^b,c^	26.71 ± 1.17^b^	4.68 ± 0.09^m^	11.22 ± 0.81^e,f^
C1	78.13 ± 0.09^h^	46.28 ± 0.30^f,g,h^	17.60 ± 0.12^e,f^	11.10 ± 0.63^g,h^	25.20 ± 0.81^b,c^
C2	86.39 ± 1.35^c,d^	58.49 ± 0.58^b,c^	26.51 ± 1.21^b^	19.15 ± 0.22^c^	4.41 ± 1.10^g,h^
C3	87.94 ± 0.05^b,c^	81.55 ± 0.35^a^	5.88 ± 0.04^i^	5.71 ± 0.04^l,m^	7.01 ± 0.14^f,g^
C4	82.52 ± 1.67^efg^	54.49 ± 0.46^cde^	22.03 ± 0.30^c^	17.00 ± 0.20^c^	8.92 ± 0.76^f,g^
C5	79.92 ± 1.33^g,h^	37.38 ± 0.61^j^	19.48 ± 0.08^d,e^	10.91 ± 0.07^g,h,i^	32.38 ± 1.00^a^
C6	83.98 ± 3.55^d,e,f^	65.78 ± 1.83^b^	34.54 ± 1.22^a^	12.23 ± 0.09^f^	8.69 ± 1.21^f,g^
C7	94.87 ± 0.09^a^	57.87 ± 0.93^b,c^	9.61 ± 0.08^h^	7.39 ± 0.09^k,l^	25.17 ± 0.57^b,c^
F1	86.36 ± 0.33^c,d^	59.79 ± 0.18^b^	22.05 ± 0.06^c^	22.83 ± 0.28^b^	0.00 ± 0.00^i^
F2	82.22 ± 0.67^e,f,g^	50.42 ± 0.44^d,e,f,g^	10.91 ± 0.06^h^	11.92 ± 0.07^f,g^	26.75 ± 0.48^b^
F3	80.18 ± 1.00^g,h^	47.22 ± 1.61^e,f,g,h^	11.01 ± 0.07^h^	6.52 ± 0.03^l^	35.37 ± 1.93^a^
F4	82.09 ± 0.98^e,f,g^	48.53 ± 0.40^e,f,g^	10.67 ± 0.04^h^	16.42 ± 0.17^c,d^	24.38 ± 0.42^b,c^
F5	88.05 ± 0.06^b,c^	80.00 ± 0.35^a^	1.79 ± 0.02^j^	8.02 ± 0.09^k,l^	10.39 ± 0.10^e,f,g^
F6	84.23 ± 0.06^d,e^	50.01 ± 0.08^d,e,f,g^	25.07 ± 0.09^b^	17.67 ± 0.15^c^	7.24 ± 0.15^f,g^

*Note:* *Different letters in the same column means significant difference (*p* < 0.05). Pouches flavored as beef (B), chicken (C), or fish (F).

To enable meaningful comparison across products with variable moisture content, proximate composition was also calculated on a DMB (Table 2). Protein content varied significantly from 37.38% (C5) to 89.97% (B4, *p* < 0.05). According to AAFCO (2023), crude protein and fat levels should meet minimum nutritional requirements, as well, reflect the biological availability of the ingredients.

Lipid or fat content also showed significant disparity, varying from 1.79% (F5) to 34.54% (C6, *p* < 0.05). Several fish and beef products clustered in the lowest fat content with less than 11% fat (Table 2). Ash content, indicative of total mineral matter, presented a more than tenfold significant range (B7: 4.68% to B5: 25.30%, *p* < 0.05). Bone and meat meal are commonly used as pet food ingredients (Simonova et al. 2020). Thus, the relatively elevated ash contents in some products may indicate mineral enrichment or inclusion of bone‐derived materials, which warrants attention to the bioavailability and balance of essential and potentially excessive minerals (Gharibzahedi and Jafari 2017). Carbohydrate levels, where present, were generally low but also varied significantly, with a maximum of 35.37% (F3) to 0.00% (B4 and F1, *p* < 0.05). Wet pet food usually does not require starch for structural integrity because their manufacturing process differs fundamentally from that of dry kibble, explaining the low amount of carbohydrate (Sgorlon et al. 2022). However, the elevated carbohydrate content observed in some pouches (C5: 32.38%, F2: 26.75%, C1: 25.20%, C7: 25.17%, and F4: 24.38%) may be of physiological relevance given the feline species’ limited capacity for carbohydrate utilization.

Collectively, these findings underscore a marked heterogeneity in the nutritional profiles of wet cat foods in the Brazilian market. The significant variability is not systematically linked to primary flavor or marketing claims, but rather points to substantial differences in raw material selection, formulation, and processing parameters (Ali et al. 2025). This variability has direct implications for the nutritional adequacy and consistency of products intended for feline consumption (Pedrinelli et al. 2019).

### 3.2 pH, Oxidative Stability and Toxic Trace Minerals in Commercial Wet Cat Food

The analysis of pH, protein (DNPH), and lipid oxidation (TBARS) revealed significant differences across product categories (*p* < 0.05, Table 3), with important implications for product stability and safety. Firstly, the products pH values varied widely from 5.78 (C7) to 7.50 (F1, *p* < 0.05). However, there was no trend on pH values regarding the different flavors. The pH of wet pet food is determined by the inherent properties of its meat ingredients—such as muscle fiber type and natural buffering capacity—and is subsequently modified by the addition of technological additives. Furthermore, this pH can decrease over time due to chemical degradation processes like fat hydrolysis and protein autolysis during storage (Ali et al. 2025; Sgorlon et al. 2022).

**Table 3:** Commercial wet cat food pH), oxidative stability measured thiobarbituric acid reactive substances (TBARs) for lipid oxidation (mg malondialdehyde (MDA)/kg dry matter basis) and by 2,4‐dinitrophenylhydrazine (DNPH) for protein oxidation (carbonyl/mg protein dry matter basis), and total mercury concentration (Hg: mg/kg dry matter basis).

Sample	pH	TBARs (mg MDA/kg)	DNPH (carbonyl/mg)	Hg (mg/kg)
**B1**	7.20 ± 0.18^b,c^	5.88 ± 0.81^b,c,d^	0.28 ± 0.04^d,e,f^	0.06 ± 0.11^c^
**B2**	6.65 ± 0.09^d^	6.88 ± 0.78^b^	0.65 ± 0.05^a,b,c,d^	0.05 ± 0.06^c^
**B3**	6.40 ± 0.30^d^	1.09 ± 0.14^f,g^	0.77 ± 0.07^a,b,c^	0.06 ± 0.06^c^
**B4**	5.98 ± 0.09^e,f^	1.03 ± 0.28^g^	0.64 ± 0.04^a,b,c,d^	0.19 ± 0.19^c^
**B5**	7.20 ± 0.02^b,c^	7.84 ± 3.13^b^	0.47 ± 0.06^c,d,e,f^	0.25 ± 0.32^b,c^
**B6**	6.952 ± 0.05^c^	1.20 ± 0.23^f,g^	0.16 ± 0.03^e,f^	0.74 ± 0.75^b^
**B7**	6.54 ± 0.03^d^	2.13 ± 0.25^e,f,g^	0.14 ± 0.03^f^	0.00 ± 0.00^c^
**C1**	6.53 ± 0.15^d^	2.75 ± 0.37^d,e,f^	0.91 ± 0.42^a,b^	0.00 ± 0.00^c^
**C2**	7.01 ± 0.03^c^	9.49 ± 2.74^b^	0.58 ± 0.02^a,b,c,d,e^	0.00 ± 0.00^c^
**C3**	5.97 ± 0.05^e,f^	1.33 ± 0.17^f,g^	0.15 ± 0.03^e,f^	0.00 ± 0.00^c^
**C4**	5.92 ± 0.05^e,f^	3.67 ± 0.90^c,d,e^	1.01 ± 0.08^a^	0.00 ± 0.00^c^
**C5**	7.02 ± 0.04^c^	5.08 ± 1.73^b,c,d^	0.57 ± 0.03^a,b,c,d,e,f^	0.00 ± 0.00^c^
**C6**	7.35 ± 0.12^a,b^	2.19 ± 0. 30^e,f,g^	0.47 ± 0.52^b,c,d,e,f^	0.00 ± 0.00^c^
**C7**	5.78 ± 0.08^f^	13.06 ± 4.88^c,d,e^	0.14 ± 0.03^e,f^	0.00 ± 0.00^c^
**F1**	7.50 ± 0.06^a^	2.64 ± 0.22^e,f,g^	0.43 ± 0.49^c,d,e,f^	0.00 ± 0.00^c^
**F2**	7.38 ± 0.11^a,b^	12.54 ± 1.20^a^	0.15 ± 0.02^e,f^	0.00 ± 0.00^c^
**F3**	6.05 ± 0.09^e^	5.45 ± 1.99^b,c^	0.35 ± 0.40^c,d,e,f^	0.00 ± 0.00^c^
**F4**	6.60 ± 0.01^d^	2.90 ± 0.46^e,f,g^	0.15 ± 0.02^e,f^	0.00 ± 0.00^c^
**F5**	7.09 ± 0.07^c^	1.00 ± 0.17^g^	0.18 ± 0.08^e,f^	1.34 ± 0.25^a^
**F6**	6.46 ± 0.02^d^	2.16 ± 0.19^e,f,g^	0.15 ± 0.05^e,f^	0.00 ± 0.00^c^

*Note:* *Different letters in the same column means significant difference (*p* < 0.05). Pouches flavored as beef (B), chicken (C) or fish (F).

The oxidative stability was assessed as an indicator of product freshness and nutritional quality, indicating both lipid and protein damage. Lipid oxidation levels, expressed on DMB to enable comparison across products with variable moisture content, showed significant differences, with values ranging from 1.00 ± 0.17 mg MDA/kg DMB (F5) to 13.06 ± 4.88 mg MDA/kg DMB (C7, *p* < 0.05). The oxidative susceptibility of chicken‐based formulations aligns with prior observations by Santana Neto et al. (2021), who observed the generation of lipid oxidation compounds during storage. As reported, lipid oxidation in wet cat food occurs because poultry fats are particularly prone to peroxidation due to their high content of polyunsaturated fatty acids (Cartoni Mancinelli et al. 2021). However, statistical analysis highlighted that the most oxidized individual sample on a DMB was C7 (13.06 mg MDA/kg DMB), a chicken‐flavored product with extremely high moisture (94.87%), followed by F2 (12.54 mg MDA/kg DMB).

The unexpectedly high lipid oxidation in beef‐flavored wet foods is primarily due to formulations containing little actual beef (Table 1), instead relying on oxidation‐prone poultry by‐products, heme‐iron, and pro‐oxidant metals like aluminum, compounded by flexible labeling laws (Galera et al. 2019). Furthermore, the high moisture content of wet food accelerates hydrolytic rancidity through autolysis, releasing vulnerable free fatty acids and overwhelming the product's antioxidant defenses, especially if processing damaged these protective systems (Ali et al. 2025).

On the other hand, samples B3 (1.09 mg MDA/kg DMB), B4 (1.03 mg MDA/kg DMB), B6 (1.20 mg MDA/kg DMB), C3 (1.33 mg MDA/kg DMB), and F5 (1.00 mg MDA/kg DMB) presented the lowest TBARS values, indicating significantly better lipid preservation. This suggests that susceptibility to lipid oxidation is highly product‐specific and not intrinsically linked to the primary protein source, but rather to factors like ingredient composition, processing, and antioxidant protection. Lipid oxidation reduces nutritional value, contributes to off‐flavors, decreases palatability, and production of cytotoxic aldehydes such as malondialdehyde (MDA), which may have long‐term health implications (Gharby et al. 2025).

In respect to protein oxidation, carbonyl content achieved the highest content in sample C4 (1.01 carbonyl/mg, *p* < 0.05), while the lowest values were found in several products across all flavors (< 0.20 carbonyl/mg, Table 3). Notably, fish‐flavored wet catfish food presented the lowest values, while chicken samples present the higher levels. This indicated processing conditions and the specific protein matrix, rather than being exclusive to a single meat type. Protein carbonylation is a marker of advanced oxidation and is associated with diminished protein quality and digestibility (Zhang et al. 2025). According to Lu et al. (2025), oxidative modification of amino acid side chains compromises protein functionality and bioavailability. The elevated values observed in certain products might reflect higher myoglobin content or exposure to thermal processing, which enhances oxidation via Maillard‐type reactions (Lu et al. 2025). These effects are particularly relevant for aging animals with increased oxidative sensitivity and dietary protein needs (Ioannidou et al. 2025).

To enable comparison across products with variable moisture content, mercury concentrations were also calculated on DMB, which ranged from not detectable to 1.34 (F5, *p* < 0.05). Sample B6 showed an intermediate value of 0.74 mg/kg DMB, while all other samples had Hg concentrations below 0.25 mg/kg DMB or were not detectable. Hg accumulation in fish‐based pet foods is a recognized concern, especially due to the potential presence of methylmercury (Sires et al. 2019), which is neurotoxic even at low doses (Charkiewicz et al. 2025).

As well, studies in cats have established a no‐observed‐adverse‐effect level (NOAEL) for methylmercury of 20 µg/kg body weight per day, with clinical signs of toxicity appearing at 46 µg/kg/day (Charbonneau et al. 1976). Assuming a typical adult cat weight of 4 kg and a daily wet food intake of 500 g, the NOAEL corresponds to a daily intake of 80 µg of Hg. Among the analyzed products, the highest mercury concentration was found in sample F5 (equivalent to 0.16 mg/kg wet basis), which would result in a daily intake of 80 µg Hg/day, as the NOAEL. Fortunately, all other samples contained ≤ 0.04 mg/kg Hg on a wet basis (≤ 0.25 mg/kg DMB for most samples, with B6 at 0.74 mg/kg DMB), corresponding to a daily intake of ≤ 20 µg Hg/day, which is only 25% of the NOAEL. Therefore, with the exception of the highest sample, the Hg levels detected in most commercial wet cat foods fall below the threshold associated with long‐term adverse effects. Nonetheless, given the potential for cumulative exposure from multiple dietary sources and the susceptibility of kittens or geriatric cats, continued monitoring and selective sourcing of low‐trophic fish species remain advisable (Jinadasa et al. 2021).

**Figure 2:**
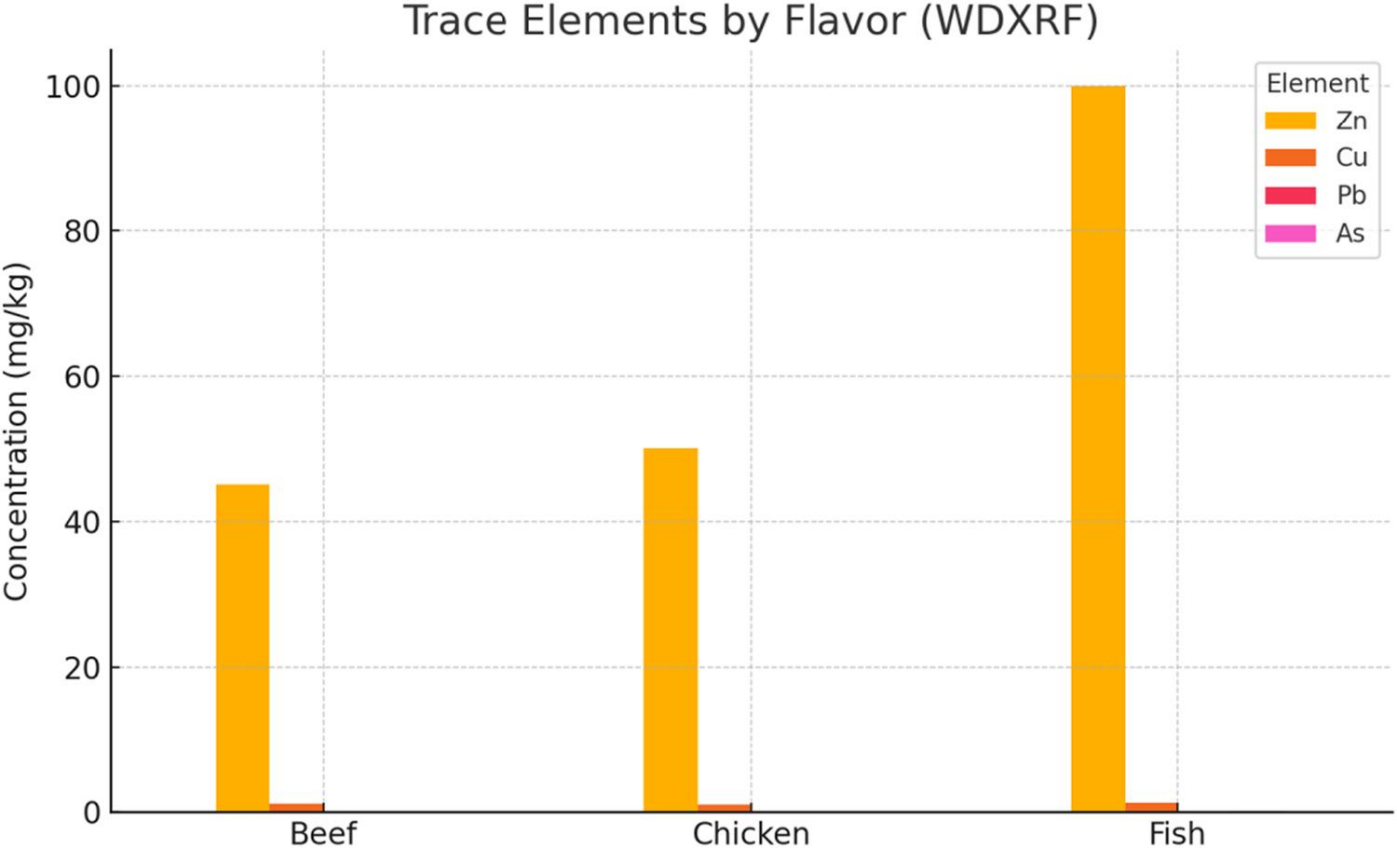
WDXRF‐based concentrations (mg/kg dry matter basis) of zinc (Zn), copper (Cu), lead (Pb) and arsenic (As) in flavored wet cat food pouches.

Strategies to mitigate risk include selective sourcing of low‐trophic fish species and batch monitoring of mercury levels in marine ingredients (Jinadasa et al. 2021). Other trace elements such as zinc (Zn), copper (Cu), lead (Pb), and arsenic (As) were determined using WDXRF, presented in Figure 2. Except from Zn, other trace elements (As and Pb) were not found or in very low levels (Cu: 0.01 mg/kg). Zn was found around 45–50 mg/kg in beef and chicken flavored wet cat food, being at least double in fish flavored products (~100 mg/kg). Trace elements play essential physiological roles but may also pose toxicity risks if unbalanced. Zinc and copper are crucial for enzymatic systems, immune function, and antioxidant defense, yet their excessive intake may interfere with the absorption of other minerals (Pedrinelli et al. 2019; Sgorlon et al. 2022; Varrà et al. 2025). The detection of arsenic, especially in fish‐based products, is consistent with the marine origin of ingredients and highlights the need for distinguishing between organic and inorganic forms. Inorganic arsenic is significantly more toxic and has been linked to carcinogenic outcomes in chronic exposures (Hackethal et al. 2023; Taylor et al. 2017). The application of WDXRF in this study offered a non‐destructive and efficient screening method, as also highlighted by Frydrych and Jurowski (2023), supporting its use in routine quality control.

The chemical analysis reveals a complex picture of product stability and safety. Significant variability in pH and oxidative markers exists within and across flavor categories, indicating that processing and formulation practices are likely more influential than the primary protein source alone. Despite being found in low levels or not detected, mercury contamination is dangerous, underscoring the need for careful sourcing and routine monitoring of food ingredients (Macías‐Montes et al. 2021; Sires et al. 2019; Zafalon et al. 2021). The observed nutritional variability reflects inconsistencies in formulation and raw material quality. High TBARs and carbonyl values suggested oxidative degradation, which may affect product palatability and safety (Sohaib et al. 2017). Although heavy metal contents were mostly within safe limits, chronic exposure remains a concern, especially for mercury and arsenic in fish‐based products (Balali‐Mood et al. 2021; Berhanu et al. 2024; Hackethal et al. 2023). Therefore, this study highlighted the need for continuous monitoring and stricter regulations for wet pet foods.

### 3.3 Biogenic Amines in Commercial Wet Cat Food

**Figure 3:**
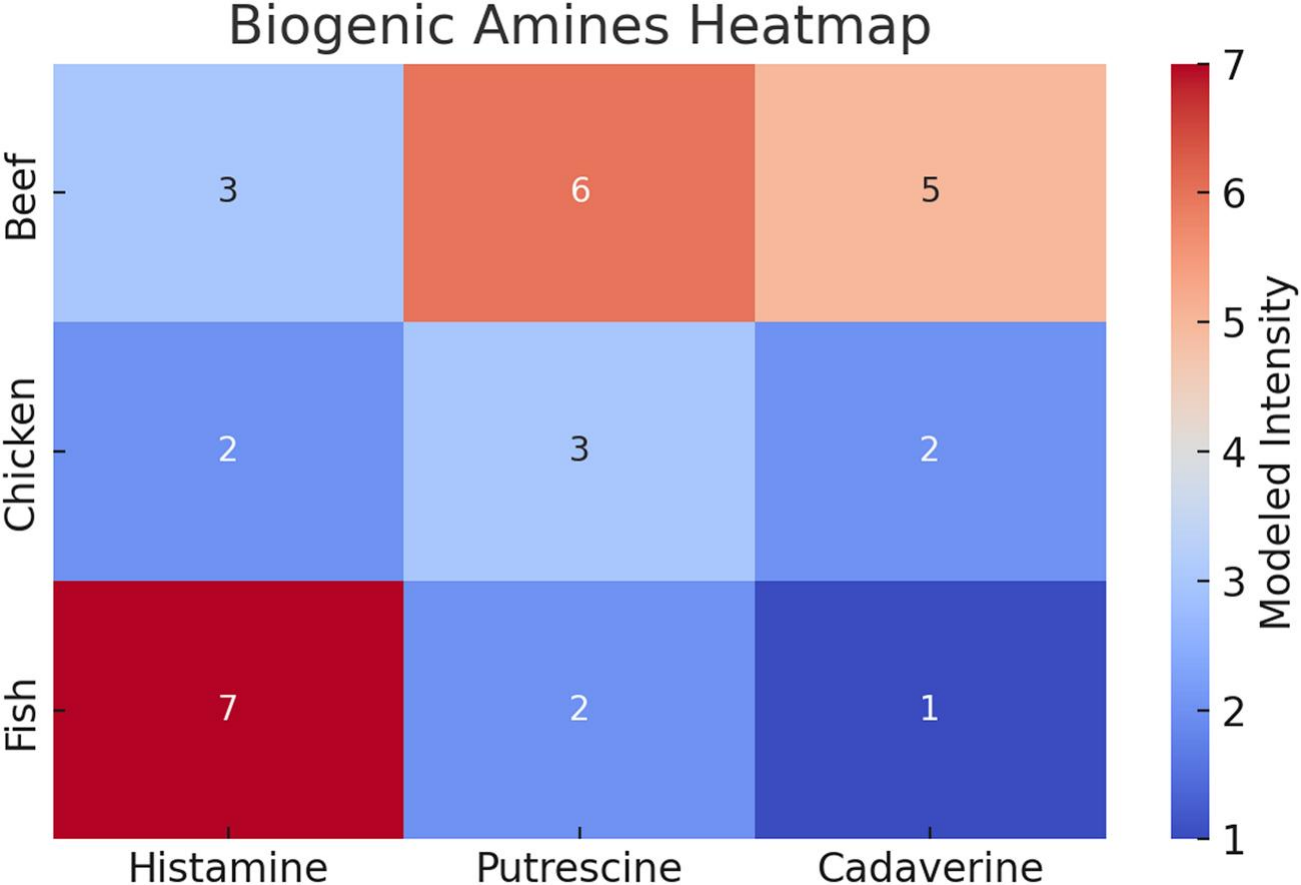
Modeled intensity of biogenic amines in flavored wet cat food.

Biogenic amines are produced through microbial decarboxylation of amino acids and serve as important indicators of hygiene and freshness (Ding and Li 2024). The heatmap presented modeled intensity values for three biogenic amines (histamine, putrescine, and cadaverine) across wet cat food flavors (Figure 3). Across flavors, histamine exhibited pronounced variation, with fish displaying the highest modeled intensity (7, corresponding to ~15.0 mg/kg), indicative of substantial histidine decarboxylation likely driven by marine bacterial activity, such as from *Morganella* sp. during processing or storage (Ding and Li 2024). In contrast, beef and chicken showed lower intensities (3 and 2, respectively, ~2.5 mg/kg), suggesting minimal accumulation, possibly attributable to lower substrate availability or stringent hygienic controls. Putrescine levels followed a different pattern, peaking in beef (intensity 6, ~7.5 mg/kg). As well, chicken registered moderate (3, ~3.5 mg/kg) and fish low (2, ~1.0 mg/kg), suggesting differences in natural polyamine content or hygienic handling among protein sources. Similarly, cadaverine was elevated in beef (5, ~7.5 mg/kg), reflecting lysine decarboxylation by *Enterobacteriaceae*, but remained low in chicken and fish (2 and 1, respectively, ~1.0 mg/kg, Figure 3).

It is important to note that polyamines such as putrescine and cadaverine are naturally present in living muscle tissue and inner organs, where they play roles in cellular growth, division, and metabolic regulation (Xuan et al. 2023). However, elevated levels can also arise from post‐mortem microbial decarboxylation of ornithine during spoilage or inadequate processing (Givanoudi et al. 2023). Therefore, the higher putrescine concentrations observed in beef‐flavored products may reflect either higher natural tissue concentrations, microbial activity, or a combination of both factors.

Flavor‐specific profiles emerged distinctly: fish was characterized by histamine dominance but low polyamines (total intensity sum: 10), posing potential risks for scombroid‐like toxicity in felines despite semi‐quantitative estimates falling below acute human thresholds; beef exhibited the highest overall amine burden (sum: 14) with balanced polyamine elevation, underscoring microbial diversity in its production chain; and chicken presented the lowest profile (sum: 7), implying superior freshness and quality control. The presence of histamine, putrescine, and cadaverine is an important quality and safety indicator in pet foods. Altafini et al. (2022) emphasized that the potential risks of repeated, low‐level (< 50 mg/kg) histamine exposure and the synergistic effects of multiple biogenic amines are not yet well understood, particularly in susceptible subpopulations such as kittens or geriatric cats. Their formation is influenced by microbial growth and improper raw material handling, reinforcing the importance of applying GMP and cold chain monitoring (Ruiz‐Capillas and Herrero 2019). Elevated putrescine and cadaverine levels are indicative of spoilage or inadequate processing (Givanoudi et al. 2023; Leelasree and Aggarwal 2022).

These disparities, robust to sensitivity checks, underscore biogenic amines as hygienic proxies in pet food, with implications for targeted interventions, such as enhanced histamine monitoring in fish formulations or preservative strategies in beef to mitigate spoilage risks and ensure safety, warranting future quantitative validation via high‐performance liquid chromatography for precise regulatory alignment.

## 4 Conclusion

This comprehensive analysis of commercial wet cat food pouches in Brazil reveals a market defined by significant and consequential variability. The study identified substantial inconsistencies in proximate composition, demonstrating that macronutrient profiles and energy density vary widely irrespective of flavor, which proved unreliable as indicators of superior quality. The assessment of chemical stability and safety identified distinct risk profiles as: (1) poultry‐flavored products showed a propensity for lipid oxidation; (2) beef‐flavored products exhibited higher levels of protein oxidation and; (3) spoilage‐related polyamines, and fish‐flavored foods were confirmed as the primary source of elevated biogenic amines like histamine. This flavor‐specific patterning of chemical hazards underscores that raw material origin is a key determinant of product integrity beyond basic nutrition.

Therefore, the findings highlighted an urgent need for a more holistic and stringent quality framework. To ensure product safety and nutritional adequacy, regulatory standards must evolve to include limits for oxidation markers and biogenic amines, while industry practices require enhancement through improved oxidative stabilization, rigorous ingredient screening, and greater formulation consistency. For veterinarians and consumers, this work underscores that informed product selection must extend beyond marketing claims to consider the combined nutritional profile, freshness indicators, and potential for chronic contaminant exposure, thereby supporting better dietary choices for feline health.

## Ethics Statement

The authors confirm that the ethical policies of the journal, as noted on the journal's author guidelines page, have been adhered to. No ethical approval was applied as this is an original article, which did not require animal subjects for the study development.

## Conflicts of Interest

The authors declare no conflicts of interest.

## Data Availability

The data that support the findings of this study are available from the corresponding author upon reasonable request.
